# Unit-based differences in compassion satisfaction, burnout, secondary traumatic stress, and turnover intention among nurses in a tertiary hospital in Abu Dhabi

**DOI:** 10.3389/fpubh.2025.1686060

**Published:** 2025-10-29

**Authors:** Zahra Mohamed, Sufian Forawi

**Affiliations:** ^1^Nursing Education, Sheikh Shakbout Medical City, Abu Dhabi, United Arab Emirates; ^2^Faculty of Education, The British University in Dubai, Dubai, United Arab Emirates

**Keywords:** compassion satisfaction, burnout, turnover intention, secondary traumatic stress, nursing workforce, Abu Dhabi, professional quality of life

## Abstract

**Background:**

Nurses are increasingly vulnerable to occupational pressures that threaten their professional well-being. These challenges are particularly pronounced in high-acuity settings, where workload, emotional demands, and environmental stressors can impact compassion satisfaction, burnout, and turnover intention. Objective: The aim of this study is to determine whether there are significant differences in Compassion Satisfaction, Burnout, secondary traumatic stress, and Turnover Intentions among nurses based on their assigned clinical area and years of nursing experience in a tertiary hospital in Abu Dhabi.

**Methods:**

A descriptive-comparative design was used. Constructs were measured using validated instruments to assess whether significant differences existed in CS, BO, STS and TI across various nurse characteristics, inferential statistical tests were conducted. A one-way Analysis of Variance (ANOVA) was used to examine differences across nurse characteristics, with significance set at *p* < 0.05. The Professional Quality of Life Scale (ProQOL, Version 5) was used to measure Compassion Satisfaction, Burnout, and Secondary Traumatic Stress, while the Turnover Intention Scale (TIS-6) assessed intention to leave.

**Results:**

The study included a total sample of *N* = 211 nurses across multiple units and designations. No statistically significant differences in Compassion Satisfaction, Burnout, Secondary Traumatic Stress, or Turnover Intention across nurse designations (Charge Nurse, Staff Nurse, Practical Nurse), although Practical Nurses reported slightly more favorable scores, particularly in burnout, which approached significance (*p* = 0.065). Turnover intention differed significantly by clinical unit [Welch’s ANOVA *F*(7, 56.3) = 2.58, *p* = 0.022], with the lowest scores in Operating Theaters/Perioperative Care and the highest in Emergency and Maternity units.

**Conclusion:**

While nurse designation and years of experience were not associated with significant differences in professional quality of life, clinical unit assignment emerged as a key factor influencing turnover intention. These findings underscore the role of organizational and environmental conditions over demographic factors in shaping nurse retention. The trend of increased burnout and turnover intention among charge nurses suggests the need for improved leadership support and resilience-building initiatives, even though it is not statistically significant. These findings highlight the importance of implementing targeted strategies such as resilience training, structured mentorship, and leadership development programs to mitigate burnout and reduce turnover intention among nurses.

## 1 Introduction

In healthcare delivery systems around the world, nurses make up over half of the workforce. According to the National Sample Survey of Registered Nurses, there were roughly 3.1 million registered nurses (RNs) in the United States alone (1, 2). Notwithstanding their crucial roles, nurses are becoming more susceptible to work-related pressures that jeopardize their professional well-being. Nurses who work in traumatic and high-acuity specialized areas are more likely to experience burnout, compassion fatigue, and secondary traumatic stress—all of which are consequences of ongoing exposure to patient pain and suffering (3, 4). Critical care units are marked by high workload, emotional exposure, and unpredictability which is associated with burnout, secondary traumatic stress, anxiety, and insomnia, and ICU cohorts typically report moderate ProQOL compassion satisfaction (5, 6).

Long-term emotional anguish, stress, and compassion fatigue might affect nurses’ job satisfaction by reducing their ability to find fulfillment in their work and raising their intention to quit. Investigating the professional quality of life and retention intentions of nurses at various care settings is becoming more and more important, as unmanaged burnout and compassion fatigue eventually contribute to turnover rates and poor patient outcomes (7). While such findings are widely reported globally, they must also be understood within the Gulf and United Arab Emirates (UAE) context. A recent cross-sectional study of UAE hospital nurses found that both work environment and job satisfaction were significant predictors of turnover intention; better work environment and higher satisfaction were associated with significantly lower intent to leave (29). These findings highlight that even modest improvements in workplace climate and satisfaction can have a substantial impact on nurse retention in the UAE context.

This study is guided by the Job Demands–Resources (JD–R) model, which suggests that high job demands (e.g., patient acuity level, understaffing, continuous emotional exposure, time pressure) increase burnout and secondary traumatic stress. On the other hand, job resources (e.g., autonomy, adequate staffing levels, standardized workflows, supportive leadership, teamwork/climate) buffer the negative effects of job demands and help sustain compassion satisfaction while reducing turnover intention (37, 38). The Professional Quality of Life (ProQOL) scale (5) assesses three domains Compassion Satisfaction (CS), Burnout (BO), and Secondary Traumatic Stress (STS). CS can buffer stress and fatigue while supporting resilience (8); BO involves emotional exhaustion, depersonalization, and reduced accomplishment (9); STS arises from direct and indirect exposure to trauma with cumulative critical incidents exposure that may reduce work engagement (10, 11). Turnover intention (TI) is a proximal indicator of attrition that is consistently associated with diminished professional quality of life (12, 13). Evidence indicates that nurses in high-acuity and fast-paced environments such as emergency departments (EDs) and intensive care units (ICUs) report higher BO, STS, and TI and lower CS than nurses in less demanding clinical units (3, 14).

Although these relationships are well described in Western settings, fewer studies examine them in the UAE. In addition, the nature of the assigned unit and the nurse’s professional experience have a significant impact on Professional Quality of Life (ProQOL) (5). Turnover Intention (TI) has become a crucial workforce outcome strongly linked to ProQOL. Several studies have shown association between nurses’ unit assignments and varying levels of compassion satisfaction (CS), burnout (BO), and TI. Fast-paced care demands, higher patient acuity, and little time for emotional processing are the reasons why units like emergency departments (EDs) and intensive care units (ICUs) routinely report higher burnout and turnover intention (3, 14). For instance, Al-Omari et al. (15) reported that nurses in Saudi Arabia and the UAE working in EDs and critical care departments were more likely to experience emotional weariness and to be less satisfied with their job than nurses working in pediatric and non-acute units. Abu Dhabi-based ICU and ED nurses also reported greater levels of stress and job unhappiness, associated with higher turnover intent (16). Among ICU nurses internationally, burnout was found to predict turnover intention—but this relationship was significantly buffered by organizational commitment (17). Conversely, nurses working in pediatric or stroke units characterized by more ongoing patient–nurse relationships and a slower pace reported higher CS and lower BO and TI, indicating better job satisfaction and emotional recovery (7).

Years of nursing experience are another important factor affecting ProQOL. Early-career nurses tend to report higher burnout and TI and lower compassion satisfaction, likely due to role transition and workload stress (29). A cross-sectional study of 900 U.S. registered nurses showed that years of practice was positively associated with compassion satisfaction and negatively with burnout and secondary traumatic stress (18). Another study among nurses in Thailand ICU settings found that those with longer practicing years were more likely to report higher compassion satisfaction (6).

The aim of this study is to determine whether there are significant differences in Compassion Satisfaction, Burnout, Secondary Traumatic Stress, and Turnover Intention among nurses based on their assigned clinical area and years of nursing experience in a hospital in Abu Dhabi. By examining these differences, the study seeks to identify how unit-specific demands and professional tenure influence nurses’ Professional Quality of Life and their intention to remain in the workforce. By examining these differences, this study provides locally relevant insights that can guide targeted retention strategies, workforce policies, and leadership interventions for high-risk clinical units.

Guided by the JD–R model and using ProQOL and TIS-6, the study addressed the following research questions:

*RQ1*: Do CS, BO, STS (ProQOL) and TI (TIS-6) differ across clinical units in a tertiary hospital in Abu Dhabi?

*RQ2*: Do CS, BO, STS (ProQOL) and TI (TIS-6) differ across years of nursing experience?

## 2 Methods

### 2.1 Study design

This cross-sectional, descriptive-comparative study aims to determine whether there are significant differences in Compassion Satisfaction, Burnout, and Turnover Intentions among nurses in Abu Dhabi based on their assigned clinical area and years of nursing experience.

### 2.2 Inclusion and exclusion criteria

The target population for this study consisted of registered nurses working across diverse specialties, including specialty units, emergency departments, operating rooms, critical care units, stroke units, and medical-surgical wards. Participants were nurses providing direct patient care within a tertiary and teaching hospital in Abu Dhabi, UAE. Nurses holding administrative positions without direct patient care responsibilities were excluded, as were nursing interns or trainees, and nursing students to maintain the focus on clinical nursing experiences and professional quality of life.

### 2.3 Sample size

The sample size was determined using G*Power software (Version 3.1.9.4) (19), based on a medium effect size (*f* = 0.25), a statistical power of 80%, and a 5% significance level (two-tailed). Based on these parameters, a minimum of 175 participants was required to detect statistically significant correlations. A non-probability convenience sampling technique was employed to recruit eligible nurses for the study. Ultimately, our achieved sample (*N* = 211) exceeded this threshold.

### 2.4 Data collection

The quantitative data for this study were collected between November 2024 and February 2025 using a cross-sectional survey design. This approach was selected to capture a snapshot of professional quality of life and turnover intentions among nurses at a specific point in time.

Data collection began with a demographic questionnaire, which gathered essential information such as age, gender, years of experience, unit assignment, and nationality. This was followed by two validated instruments: the Professional Quality of Life (ProQOL) scale, which measured compassion satisfaction, burnout, and secondary traumatic stress (5), and the Turnover Intention Scale (TIS) by Bothma & Roodt (20) which assessed the likelihood of nurses leaving their current position or the profession entirely.

A convenience sampling strategy was employed to recruit participants from various clinical areas, including emergency departments, critical care units, medical-surgical wards, specialty units, and perioperative services (4).

Prior to data collection, ethical approval was secured from the Research Ethics Committee of the British University in Dubai (BUID) and the Institutional Review Board (IRB) of the participating tertiary hospital. Additional administrative approval was obtained from the Nursing Administration Office and the Chief Nursing Officer (CNO), who authorized the distribution of the survey. The questionnaire was created using Google Forms to ensure efficient and centralized data collection while maintaining participant anonymity and confidentiality.

The survey link, accompanied by an invitation letter and an electronic informed consent statement, was distributed via an institutional email list to all eligible nurses. By proceeding with the survey, participants provided their informed consent. Upon completion of the data collection period, responses were screened for completeness and accuracy. Responses were stored on a secure, password-protected platform accessible only to the research team.

### 2.5 Measures and instruments

The measurement tools used in this study were carefully chosen for their theoretical alignment, reliability, and strong psychometric properties. Two validated instruments were employed.

The Professional Quality of Life Scale (ProQOL), developed by Stamm (5), is designed to assess both the positive and negative aspects of professional well-being. It consists of 30 self-report items rated on a five-point Likert scale (1 = never, 5 = very often), divided into three subscales: Compassion Satisfaction (CS), Burnout (BO), and Secondary Traumatic Stress (STS). The ProQOL has demonstrated excellent reliability, with Cronbach’s alpha coefficients ranging from 0.81 to 0.90 across its subscales (5). Its construct validity has been confirmed in over 200 published studies involving diverse healthcare professionals. Importantly, high scores on the Burnout and Secondary Traumatic Stress subscales have been strongly associated with increased turnover intention, underscoring its relevance in workforce retention research.

The second instrument, the Turnover Intention Scale (TIS) Scale developed and validated by Bothma and Roodt (20), is a 6-item self-report tool that measures an employee’s intention to leave their current position. Items assess the frequency of thoughts about leaving the job, job satisfaction, frustration with unmet work goals, desire for a different job, likelihood of accepting another job at the same pay, and anticipation of future workdays. Participants rate their agreement with each item on a five-point Likert scale (1 = strongly disagree, 5 = strongly agree). The TIS has demonstrated strong internal consistency in various occupational settings, including healthcare (Cronbach’s *α* ≈ 0.80–0.90), and is widely used to explore factors influencing workforce retention. Lower Turnover Intention Scale scores reflect stronger professional commitment, whereas higher scores indicate a greater likelihood of turnover. The TIS scale has been extensively utilized in hospital settings to explore factors influencing nurse retention, including job satisfaction, emotional exhaustion, and organizational support.

In the present sample of this study (*N* = 211), internal consistency was assessed using Cronbach’s alpha. The ProQOL scale demonstrated acceptable reliability (*α* = 0.687). For the Turnover Intention Scale (TIS-6), the Cronbach’s alpha was 0.517, with improved reliability observed when Item 2 was retained (*α* = 0.712).

### 2.6 Statistical analysis

To assess whether significant differences existed in CS, BO, STS, and TI across various nurse characteristics, inferential statistical tests were conducted. Statistical analyses were performed using IBM SPSS Statistics for Windows, Version 29.0 (IBM Corp., Armonk, NY, USA). One-way Analysis of Variance (ANOVA) was used to examine differences across multiple categorical variables with more than two groups. A significance level of *p* < 0.05 was set for all analyses. When assumptions for parametric testing, including normality and homogeneity of variance, were not met, Welch’s ANOVA was applied. A significance level of *p* < 0.05 was set for all analyses. Where statistically significant differences were detected, appropriate post-hoc comparisons (e.g., Games–Howell) were conducted. Assumptions for all tests were checked prior to analysis to ensure appropriateness.

### 2.7 Ethical statement

Ethical approval for this study was secured from the Research Ethics Committee at the British University in Dubai (BUID) and the Institutional Review Board (IRB) (SSMCREC-520) of the participating tertiary hospital before data collection commenced. The research complied with globally accepted protocols for studies involving human subjects, specifically the Declaration of Helsinki and the ethical framework established by the British Educational Research Association. Participants received detailed written and verbal information regarding the study’s objectives, procedures, and their rights, which included voluntary participation and the ability to withdraw at any time without consequence.

Informed consent was secured from each participant. Data were retained on a password-secured computer exclusively accessible to the principal investigator. In compliance with institutional policy and ethical research standards, all data were securely retained for 5 years post-study completion and subsequently permanently destroyed.

## 3 Results

### 3.1 Reliability analysis

Prior to conducting inferential statistics, scale reliability was examined. The ProQOL demonstrated acceptable internal consistency (*α* = 0.687). The Turnover Intention Scale (TIS) showed moderate reliability (*α* = 0.517), with improved internal consistency (*α* = 0.712) when Item 2 was retained. These values are consistent with prior reports of variability in shorter turnover intention scales.

### 3.2 Demographic characteristics of nurses

A total of 211 nurses participated in this study. Most participants had 11–15 years of experience working as a nurse (47.9%) and more than 4 years of experience at the tertiary hospital in Abu Dhabi (36.0%) as Staff Nurse (88.6%). Most participants were assigned to the Critical Care Unit (30.8%), Inpatient Medical-Surgical Units (14.7%), Oncology (10.9%), and Pediatric and Neonatal Care (10.4%) (see Table 1).

**Table 1:** Demographic characteristics of nurses (*N* = 211).

Characteristics	Frequency	Percentage
Years of experience at the hospital in Abu Dhabi		
0–3 Months	8	3.8
3 Months- 1 year	10	4.7
1–2 years	48	22.7
2–3 years	26	12.3
3–4 years	43	20.4
More than 4 years	76	36
Designation		
Charge Nurse	21	10
Staff Nurse	187	88.6
Practical Nurse	3	1.4
Years of experience working as nurse		
1–5	13	6.2
6–10	35	16.6
11–15	101	47.9
16–20	35	16.6
More than 20	27	12.8
Clinical unit currently assigned		
Critical Care Unit	65	30.8
Outpatient/ Ambulatory Care	24	11.4
Pediatric and Neonatal Care	22	10.4
Operating theaters and Perioperative care	7	3.3
Maternity and Obstetric	20	9.5
Emergency Department	19	9
Inpatient Medical- Surgical Units	31	14.7
Oncology	23	10.9

*N* = 211.

### 3.3 Comparative analysis for designation at the a tertiary hospital and study variables

Welch’s One-Way ANOVA was conducted to examine whether there were significant differences in compassion satisfaction, burnout, secondary traumatic stress, and turnover intention among different nurse designations (Charge Nurse, Staff Nurse, and Practical Nurse) (see Table 2).

**Table 2:** Welch’s one way ANOVA results comparing nurse designation and variables.

Variable	Designation	*M*	SD	95% CI Low	95% CI High	F	df₁	df₂	*p*	*η* ^2^
Compassion satisfaction	Charge nurse	36.00	4.69	30.18	41.82	1.00	2	5.02	0.430	0.285
	Staff nurse	35.35	5.21	33.87	36.83					
	Practical nurse	41.00	6.93	23.78	58.22					
Burnout	Charge nurse	26.33	3.69	21.75	30.91	4.79	2	5.27	0.065	0.645
	Staff nurse	26.04	4.16	24.86	27.22					
	Practical nurse	21.00	2.65	14.42	27.58					
Secondary traumatic stress	Charge nurse	25.43	5.77	18.27	32.59	2.97	2	5.07	0.140	0.540
	Staff nurse	22.91	6.65	21.02	24.80					
	Practical nurse	16.00	6.93	−1.22	33.22					
Turnover intention	Charge nurse	3.08	0.52	2.43	3.73	1.16	2	5.16	0.384	0.310
	Staff nurse	2.97	0.60	2.80	3.14					
	Practical nurse	2.61	0.48	1.42	3.80					

*p* < 0.05.

The analysis showed that there was no statistically significant difference in compassion satisfaction among the three nurse designations, *F*(2, 5.02) = 1.00, *p* = 0.430. Although Practical Nurses (*M* = 41.00, SD = 6.93) reported slightly higher compassion satisfaction compared to Charge Nurses (*M* = 36.00, SD = 4.69) and Staff Nurses (*M* = 35.35, SD = 5.21), this difference was not statistically significant.

The results indicated a difference that approached statistical significance in burnout among the three groups, *F*(2, 5.27) = 4.79, *p* = 0.065. While the overall significance level did not reach the conventional threshold (*p* < 0.05), the mean scores suggest that Practical Nurses (*M* = 21.00, SD = 2.65) reported lower burnout levels compared to Charge Nurses (*M* = 26.33, SD = 3.69) and Staff Nurses (*M* = 26.04, SD = 4.16).

There was no statistically significant difference in secondary traumatic stress among the groups, *F*(2, 5.07) = 2.97, *p* = 0.140. However, the descriptive statistics indicate that Practical Nurses (*M* = 16.00, SD = 6.93) reported the lowest levels of secondary traumatic stress compared to Charge Nurses (*M* = 25.43, SD = 5.77) and Staff Nurses (*M* = 22.91, SD = 6.65). The ANOVA results revealed no significant difference in turnover intention among the three groups, *F*(2, 5.16) = 1.16, *p* = 0.384. Although Practical Nurses (*M* = 2.61, SD = 0.48) reported slightly lower turnover intention scores compared to Charge Nurses (*M* = 3.08, SD = 0.52) and Staff Nurses (*M* = 2.97, SD = 0.60), the difference was not statistically significant.

Although no comparisons reached statistical significance, the effect sizes suggest moderate-to-large practical effects across most outcomes. For example, burnout (*η*^2^ = 0.645) and secondary traumatic stress (*η*^2^ = 0.540) indicate large group differences, with practical nurses reporting noticeably lower levels than staff or charge nurses. Wide confidence intervals, particularly for practical nurses due to small n, reflect uncertainty in estimates, but still suggest meaningful trends.

### 3.4 Comparative analysis for years of nursing experience and study variables

The findings indicated that there were no statistically significant differences among the experience groups for any of the variables (*p* > 0.05). The results showed that compassion satisfaction did not significantly differ across the five experience groups, *F*(4, 55.3) = 0.278, *p* = 0.891. Although mean scores ranged from 34.80 (SD = 4.77) in the 6–10 years group to 35.78 (SD = 4.63) in the more than 20 years group, these differences were not statistically significant.

Similarly, burnout levels were not significantly different among the groups, *F*(4, 54.6) = 0.492, *p* = 0.742. Nurses with 6–10 years of experience reported the highest burnout scores (*M* = 26.77, SD = 3.53), while those with more than 20 years of experience had the lowest (*M* = 25.67, SD = 3.75), though this variation was not statistically meaningful (see Table 3).

**Table 3:** Welch’s one way ANOVA for years of nursing experience and study variables.

Variable	Years of experience	*M*	SD	SE	95% CI Low	95% CI High	*F*	df₁	df₂	*p*	*η* ^2^
Compassion satisfaction	1–5 yrs	34.92	5.84	1.62	~31.7	~38.1	0.278	4	55.3	0.891	0.020
	6–10 yrs	34.80	4.77	0.81	~33.2	~36.4					
	11–15 yrs	35.73	5.57	0.55	~34.6	~36.8					
	16–20 yrs	35.51	4.91	0.83	~33.8	~37.2					
	>20 yrs	35.78	4.63	0.89	~34.0	~37.6					
Burnout	1–5 yrs	25.92	4.50	1.25	~23.3	~28.5	0.492	4	54.6	0.742	0.035
	6–10 yrs	26.77	3.53	0.60	~25.6	~27.9					
	11–15 yrs	25.83	4.15	0.41	~25.0	~26.6					
	16–20 yrs	25.97	4.87	0.82	~24.3	~27.6					
	>20 yrs	25.67	3.75	0.72	~24.3	~27.0					
Secondary traumatic stress	1–5 yrs	23.46	5.78	1.60	~20.3	~26.6	1.227	4	55.4	0.310	0.081
	6–10 yrs	24.63	5.39	0.91	~22.8	~26.5					
	11–15 yrs	22.24	6.58	0.66	~21.0	~23.5					
	16–20 yrs	23.97	8.03	1.36	~21.2	~26.7					
	>20 yrs	22.74	6.64	1.28	~20.2	~25.3					
Turnover intention	1–5 yrs	3.17	0.39	0.11	~2.9	~3.4	1.317	4	58.1	0.275	0.083
	6–10 yrs	3.08	0.52	0.09	~2.9	~3.3					
	11–15 yrs	2.91	0.60	0.06	~2.8	~3.0					
	16–20 yrs	2.99	0.68	0.12	~2.7	~3.3					
	>20 yrs	2.96	0.59	0.11	~2.7	~3.2					

*p* < 0.05.

The analysis also found no significant difference in secondary traumatic stress across experience levels, *F*(4, 55.4) = 1.227, *p* = 0.310. While nurses with 6–10 years of experience had the highest scores (*M* = 24.63, SD = 5.39), and those with more than 20 years reported the lowest (*M* = 22.74, SD = 6.64), these differences were not statistically significant.

Turnover intention scores did not significantly differ among the experience groups, *F*(4, 58.1) = 1.317, *p* = 0.275. Nurses with 1–5 years of experience had the highest turnover intention (*M* = 3.17, SD = 0.39), while those with 11–15 years of experience had the lowest (*M* = 2.91, SD = 0.60). However, these differences did not reach statistical significance.

Effect sizes were small (*η*^2^ = 0.020–0.083), suggesting that years of experience explain only a modest proportion of variance in compassion satisfaction, burnout, stress, and turnover. Secondary traumatic stress (*η*^2^ = 0.081) and turnover intention (*η*^2^ = 0.083) showed the largest effects, albeit still in the small range, indicating that nurses’ stress and intentions to leave may vary slightly depending on experience level.

### 3.5 Comparative analysis for clinical unit currently assigned and study variables

The results of the Welch’s One-way ANOVA for compassion satisfaction showed no statistically significant difference across nursing units, *F*(7, 54.6) = 1.463, *p* = 0.200. Mean scores for compassion satisfaction ranged from 34.48 (SD = 4.88) for nurses in Inpatient Medical-Surgical Units to 37.42 (SD = 4.31) for those in Outpatient/Ambulatory Care (see Table 4).

**Table 4:** Welch’s one way ANOVA for clinical unit currently assigned and study variables.

Variable	Unit/group	*M*	SD	SE	95% CI Low	95% CI High	*F*	df₁	df₂	*p*	*η* ^2^
Compassion satisfaction	Critical care	34.51	5.64	0.70	~33.1	~35.9	1.463	7	54.6	0.200	0.158
	Outpatient/Ambulatory	37.42	4.31	0.88	~35.7	~39.1					
	Pediatric & Neonatal	36.14	4.75	1.01	~34.1	~38.2					
	Operating/Perioperative	36.71	6.13	2.32	~32.1	~41.3					
	Maternity & Obstetric	37.00	4.46	1.00	~35.0	~39.0					
	Emergency Department	35.63	7.04	1.62	~32.3	~39.0					
	Inpatient Medical-Surgical	34.48	4.88	0.88	~32.7	~36.2					
	Oncology	35.26	3.66	0.76	~33.8	~36.7					
Burnout	Critical care	26.88	4.20	0.52	~25.8	~27.9	1.231	7	55.3	0.302	0.134
	Outpatient/Ambulatory	24.88	3.29	0.67	~23.5	~26.2					
	Pediatric & Neonatal	25.14	3.86	0.82	~23.5	~26.8					
	Operating/Perioperative	24.57	3.60	1.36	~21.9	~27.2					
	Maternity & Obstetric	25.30	3.34	0.75	~23.8	~26.8					
	Emergency Department	26.21	4.49	1.03	~24.1	~28.3					
	Inpatient Medical-Surgical	26.65	5.49	0.99	~24.7	~28.6					
	Oncology	25.48	2.95	0.62	~24.2	~26.7					
Secondary traumatic stress	Critical care	23.78	6.62	0.82	~22.1	~25.5	0.990	7	54.6	0.448	0.112
	Outpatient/Ambulatory	21.13	5.74	1.17	~18.8	~23.5					
	Pediatric & Neonatal	22.05	6.45	1.37	~19.3	~24.8					
	Operating / Perioperative	20.14	7.36	2.78	~14.7	~25.6					
	Maternity & Obstetric	23.25	5.18	1.16	~21.0	~25.5					
	Emergency Department	23.58	7.38	1.69	~20.2	~27.0					
	Inpatient Medical-Surgical	24.81	8.12	1.46	~21.9	~27.7					
	Oncology	21.96	5.57	1.16	~19.6	~24.3					
Turnover Intention	Critical Care	3.03	0.59	0.07	~2.9	~3.2	2.580	7	56.3	0.022*	0.244
	Outpatient / Ambulatory	2.93	0.64	0.13	~2.7	~3.2					
	Pediatric & Neonatal	2.77	0.50	0.11	~2.5	~3.0					
	Operating/Perioperative	2.55	0.36	0.13	~2.3	~2.8					
	Maternity & Obstetric	3.08	0.70	0.16	~2.8	~3.4					
	Emergency Department	3.15	0.60	0.14	~2.9	~3.4					
	Inpatient Medical-Surgical	3.07	0.53	0.10	~2.9	~3.3					
	Oncology	2.80	0.54	0.11	~2.6	~3.0					

*p* < 0.05.

For burnout, the analysis also indicated no significant difference among the nursing units, *F*(7, 55.3) = 1.231, *p* = 0.302. The highest mean burnout score was observed in Inpatient Medical-Surgical Units (*M* = 26.65, SD = 5.49), while the lowest was found in Outpatient/Ambulatory Care (*M* = 24.88, SD = 3.29).

Similarly, secondary traumatic stress did not significantly differ across nursing units, F(7, 54.6) = 0.990, *p* = 0.448. The lowest mean score was observed among nurses in Operating Theaters and Perioperative Care (*M* = 20.14, SD = 7.36), while the highest was in Inpatient Medical-Surgical Units (*M* = 24.81, SD = 8.12).

Turnover intention significantly differed across clinical units, *F*(7, 56.3) = 2.580, *p* = 0.022. The highest scores were observed in the Emergency Department (*M* = 3.15, SD = 0.60) and Maternity and Obstetric units (*M* = 3.08, SD = 0.70), while the lowest were in Operating Theaters and Perioperative Care (*M* = 2.55, SD = 0.36).

*Post-hoc* pairwise comparisons were conducted to identify which clinical units differed on turnover intention. Given the use of Welch’s ANOVA, Games–Howell tests were used, with Benjamini–Hochberg (BH) correction for multiple comparisons; Tukey’s HSD was also examined because Levene’s test did not indicate heterogeneity of variances (*p* = 0.599). The largest unadjusted mean differences were observed between the Emergency Department and Maternity & Obstetrics [ΔM = 0.60, 95% CI (0.09, 1.12), *p* = 0.021] and between Operating Theaters/Perioperative Care and Maternity & Obstetrics [ΔM = 0.54, 95% CI (0.01, 1.06), *p* = 0.041]. However, no pairwise contrasts remained significant after multiple-comparison correction (BH-adjusted *p* ≥ 0.093), and Tukey’s HSD likewise indicated no significant pairwise differences. Descriptively, the Emergency Department showed the highest mean turnover intention (*M* = 3.15), whereas Operating Theaters/Perioperative Care showed the lowest (*M* = 2.55). These findings suggest that, while descriptive trends indicate higher turnover intention in the Emergency Department and Maternity & Obstetrics, no single pair of units differed significantly after adjustment.

## 4 Discussion

### 4.1 Comparative analysis for designation at a tertiary hospital and study variables

The analysis showed no statistically significant difference in compassion satisfaction, burnout, secondary traumatic stress, and turnover intention among the three nurse designations- Charge Nurses, Staff Nurses, and Practical Nurses (all *p* > 0.05). Although effect sizes suggested practical nurses reported somewhat lower burnout and stress (*η*^2^ = 0.645 for and 0.540 respectively). In contrast to our results, Shah et al. (21) reported high burnout among U.S. nurses overall but did not report designation-specific effects, suggesting that systemic factors may outweigh roles. Similarly, a systematic review of nurse managers highlighted greater emotional strain in leadership positions but not consistently in charge roles (39). Regional studies in Oman and Saudi Arabia reported high burnout prevalence across nurses without clear designation-based variation (40, 41). These findings indicate that in the UAE context, organizational conditions such as workload, staffing, and leadership support may be more decisive determinants of professional quality of life than designation alone.

This finding aligns with the notion that role-based distinctions in nursing may be less pronounced in practice, particularly in high-acuity environments such as critical care, where overlapping responsibilities and shared exposure to patient care stressors are common (22). The lack of notable differences may be attributed to the uniformity of working conditions, which may lessen the variability in psychological outcomes between roles.

The possible impact of personal coping strategies, resilience, personality qualities, and support systems all of which can differ more within groups than between them is another possibility. Prior studies indicate that intrapersonal factors can attenuate or exacerbate the effects of job demands across nursing roles (23). Although they were not taken into account in our analysis, these individual characteristics may lessen or increase the effect of job classification on professional quality of life.

### 4.2 Comparative analysis for years of nursing experience and variables

The results show no statistically significant differences in compassion satisfaction, burnout, secondary traumatic stress, or turnover intention amongst the different experience groups, whether expressed as years of experience at the tertiary hospital or total years of nursing practice. Although some effect sizes were observed, these differences did not reach significance.

This aligns with prior studies showing mixed or no significant differences in burnout, compassion fatigue, and job satisfaction when stratifying by years of experience, especially in high-acuity settings (24, 25). In contrast, some studies have noted protective effects of experience, suggesting that more experienced nurses may develop adapting coping mechanisms, whereas less experienced nurses benefit from close team support (23, 26). These findings suggest that in high-acuity settings, environmental demands such as workload, staffing, and leadership support may exert greater influence on professional quality of life than years of experience alone (27, 28).

### 4.3 Comparative analysis for clinical unit currently assigned and variables

The results of the Welch’s ANOVA for compassion satisfaction, burnout and traumatic stress showed no statistically significant difference across nursing units suggesting that unit type alone may not be a decisive determinant of these outcomes. Maslach and Jackson (42) described burnout as emotional weariness, depersonalization, and decreased personal achievement. Large-scale studies have shown that staffing adequacy, shift scheduling, and resource availability are significant predictors of burnout (27). Recent regional evidence also links persistent understaffing, frequent overtime, and high patient acuity as conditions common in UAE tertiary hospitals with elevated burnout scores (29). These high-demand environments can perpetuate emotional fatigue and erode resilience, even among experienced staff (24). Conversely, ambulatory and perioperative care units often benefit from more predictable caseloads, structured scheduling, and standardized protocols, factors associated with reduced emotional exhaustion and improved work-life balance (30, 31).

These results signal the importance of tailored burnout-prevention strategies, especially in units where nurses work in sustained crisis mode. Efforts should include staffing adjustments, scheduled rest breaks, emotional resilience workshops, and managerial support systems.

### 4.4 Limitations of the work

Although this study provides valuable insights into unit-based differences in compassion satisfaction, burnout, secondary traumatic stress, and turnover intention among hospital nurses in Abu Dhabi, several limitations should be noted. First, using a cross-sectional methodology makes it more challenging to demonstrate a causal relationship between turnover intention and ProQOL components (secondary traumatic stress, burnout, and compassion satisfaction). Second, the study employed a non-probability convenience sampling technique, this may have introduced selection bias which limits the generalizability of the findings beyond the specific hospital setting. Future research should consider using probability-based sampling to enhance external validity. Despite being sizable enough for statistical analysis, the sample might not fairly represent the diversity of nurses in other Abu Dhabi healthcare facilities or the wider United Arab Emirates.

Third, while the achieved sample size met the minimum requirement determined through *a priori* power analysis using G*Power, ensuring adequate statistical power, the possibility of non-response bias cannot be excluded. Nurses who did not participate may differ systematically in terms of workload, stress levels, or turnover intentions, which could influence the results.

Fourth, conducting the study within a single institution limits the ability to account for variations in organizational culture, staffing models, and resource availability across the UAE’s diverse healthcare sectors, including public, private, and semi-government facilities. Fifth, due to the fact that the questionnaires were self-reported online, there might have been response bias from participants who, depending on their own opinions or social desirability, may have inflated or underestimated their stress, satisfaction, or intention to leave. Sixth, the TIS-6 demonstrated moderate reliability (*α* = 0.517) in our sample, although this improved to α = 0.712 when Item 2 was retained. Shorter scales such as TIS-6 are known to show variable reliability, particularly in more homogeneous occupational groups, and single items may disproportionately influence internal consistency. This pattern has been noted in previous psychometric research on turnover intention scales (20). Future studies in the UAE nursing context may benefit from confirmatory factor analysis, item refinement, or alternative measures to strengthen reliability lastly, this study did not account for organizational elements like workload ratios, leadership ideologies, and institutional support that may also influence turnover intention, even if validated tools like ProQOL and TIS were used.

## 5 Implications for nursing management

The results of this study highlight the need for workplace interventions tailored to meet the unique requirements of clinical units rather than depending only on demographic factors such as nurses’ designations or years of experience. Notably, the prevalence rates observed in the present study mirror international trends, where turnover intention remains a significant concern in high-stress clinical environments, including emergency departments (32, 33). Within the Gulf region, similar challenges have been documented, with burnout frequently attributed to workload intensity, staffing limitations, and the emotional demands of care delivery (34). This alignment with both global and regional evidence reinforces the urgency of adopting organizational measures that actively support nurse well-being, foster professional engagement, and reduce attrition risk. Recent work highlights the value of structured retention strategies and workplace interventions aimed at improving morale and resilience (35), which could be adapted to the context of Abu Dhabi’s healthcare system.

While compassion satisfaction, burnout, and turnover intention did not differ statistically significantly according to years of experience or designation; a notable variation in turnover intention was observed between clinical units. This reinforces evidence that organizational and environmental characteristics, such as workload intensity, emotional demands, and resource availability, may exert greater influence on nurses’ intent to leave than personal demographics (36, 43, 44).

Furthermore, the trend of increased burnout and turnover intention among charge nurses suggests the need for improved leadership support and resilience-building initiatives, even though it is not statistically significant. Unit-specific strategies, such as enhanced staffing flexibility in emergency and maternity units, structured mentorship for charge nurses, and access to psychological support services, may help reduce burnout and improve retention. In order to improve retention and cultivate compassion satisfaction among nurses in Abu Dhabi, healthcare managers and nurse leaders should prioritize enhancing work conditions, encouraging supportive leadership, and putting mental health resources into place.

## 6 Conclusion

This study examined unit-based differences in compassion satisfaction, burnout, secondary traumatic stress, and turnover intention among nurses at a hospital in Abu Dhabi. While no statistically significant differences, which looked at the effects of nurse designation, years of experience, and clinical unit assignment on their levels of burnout, secondary traumatic stress, compassion satisfaction, and turnover intention. No statistically significant differences were found in any of the professional quality of life variables between nurse designations or years of experience. However, there was a notable variation in the intention to quit among clinical units, indicating that the work environment may have a greater association with nurses’ intention to leave than role or tenure. Interestingly, charge nurses scored higher on burnout and turnover intention than practical nurses, while the differences were not statistically significant. Nurses in perioperative units reported the lowest turnover intention, whereas those in emergency and maternity units reported the highest, likely due to the intense demands and emotional labor associated with these settings.

## Data Availability

The raw data supporting the conclusions of this article will be made available by the authors, without undue reservation.
